# Mobile caseous mitral annular calcification as a cause of recurrent cardioembolic stroke: a case report

**DOI:** 10.1093/ehjcr/ytag471

**Published:** 2026-06-25

**Authors:** Elsa Hoti, Jorge Sierra, Georgios Tzimas, Francesc Carbo-Descamps, Sarah Hugelshofer

**Affiliations:** University of Lausanne, University Hospital of Lausanne, Rue du Bugnon 46, Lausanne 1011, Switzerland; Department of Cardiac Surgery, University Hospital of Lausanne, Rue du Bugnon 46, Lausanne 1011, Switzerland; Department of Cardiology, University Hospital of Lausanne, Rue du Bugnon 46, Lausanne 1011, Switzerland; Institute of Pathology, University Hospital of Lausanne, Rue du Bugnon 46, Lausanne 1011, Switzerland; Department of Cardiology, University Hospital of Lausanne, Rue du Bugnon 46, Lausanne 1011, Switzerland

**Keywords:** Caseous mitral annular calcification, Cardioembolic stroke, Mitral valve disease, Multimodality imaging, Case report

## Abstract

**Background:**

Caseous mitral annular calcification (CMAC) is a rare variant of mitral annular calcification that may occasionally lead to systemic embolization.

**Case Summary:**

We report the case of a 68-year-old woman with cardiovascular risk factors and a heterozygous factor V Leiden mutation presenting with recurrent embolic strokes. Initial investigations in 2021 revealed a posterior mitral annular mass with central echolucency on transoesophageal echocardiography, consistent with CMAC, associated with a small thrombus. Anticoagulation was initiated, and serial imaging demonstrated a stable lesion over several years. In 2025, the patient developed recurrent multifocal cerebral infarctions. Repeat imaging showed progression of the mass with a newly mobile atrial component, indicating high embolic risk. Multimodality imaging, including echocardiography and cardiac computed tomography, confirmed the diagnosis and excluded differential diagnoses such as tumour or infective endocarditis. The patient underwent mitral valve replacement with complete resection of the lesion. Histopathological examination showed signs of advanced CMAC. Postoperative recovery was uneventful, with no recurrence of embolic events.

**Discussion:**

This case highlights the dynamic nature of CMAC and underscores the importance of close follow-up and timely surgical intervention in the presence of recurrent embolization or high-risk structural abnormalities.

Learning points

- Caseous mitral annular calcification is a rare but under-recognized cause of cardioembolic stroke.
- The development of a mobile component indicates increased embolic risk and may warrant surgical intervention.

## Introduction

Caseous mitral annular calcification (CMAC) is a rare variant of mitral annular calcification (MAC), characterized by a rounded, hyperechogenic mass, typically located within the posterior mitral annulus, with a translucent centre on echocardiography corresponding to liquefied necrosis. The prevalence of CMAC on imaging is estimated at less than 0.1% in the general population and 0.64% among patients with MAC.^1,2^ Although usually detected incidentally, CMAC may occasionally be associated with clinically significant complications, including systemic embolization and valvular dysfunction.^2,3^

We report a case of CMAC complicated by recurrent embolic strokes, undergoing surgical cure, highlighting the diagnostic challenges and clinical implications of this uncommon entity.

## Summary figure

**Figure:**
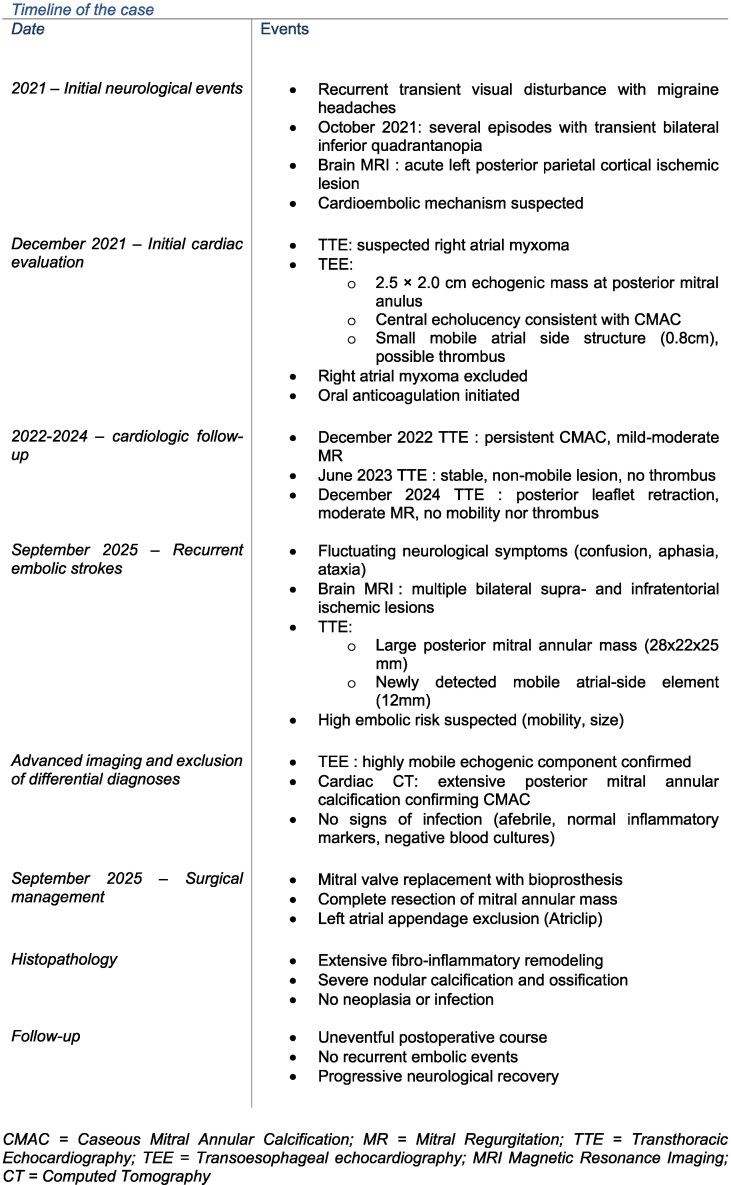


## Case presentation

A 68-year-old woman with hypertension, hypercholesterolaemia, and heterozygous factor V Leiden mutation was urgently admitted in September 2025 for fluctuating neurological symptoms: transient confusion, aphasia, and left-arm ataxia. Her neurological history dated back to 2021, when she presented with transient visual deficits and migraine-like headaches. MRI showed an acute ischaemic lesion in the left posterior parietal region, suggestive of cardioembolic source. Transthoracic echocardiography (TTE) revealed a left atrial mass with initial suspicion for myxoma according to the consultant cardiologist. However, transoesophageal echocardiography (TEE) identified a 2.5 cm × 2.0 cm rounded hyperechogenic lesion with translucent centre within the posterior mitral annulus, which was consistent with caseous mitral annular calcification (CMAC), but excluded patent foramen ovale and left atrial appendage thrombus. A small thrombus was attached to the CMAC, and moderate mitral regurgitation was induced by slightly restricting the posterior leaflet. Anticoagulation therapy with acenocoumarol was initiated.

**Figure 1:**
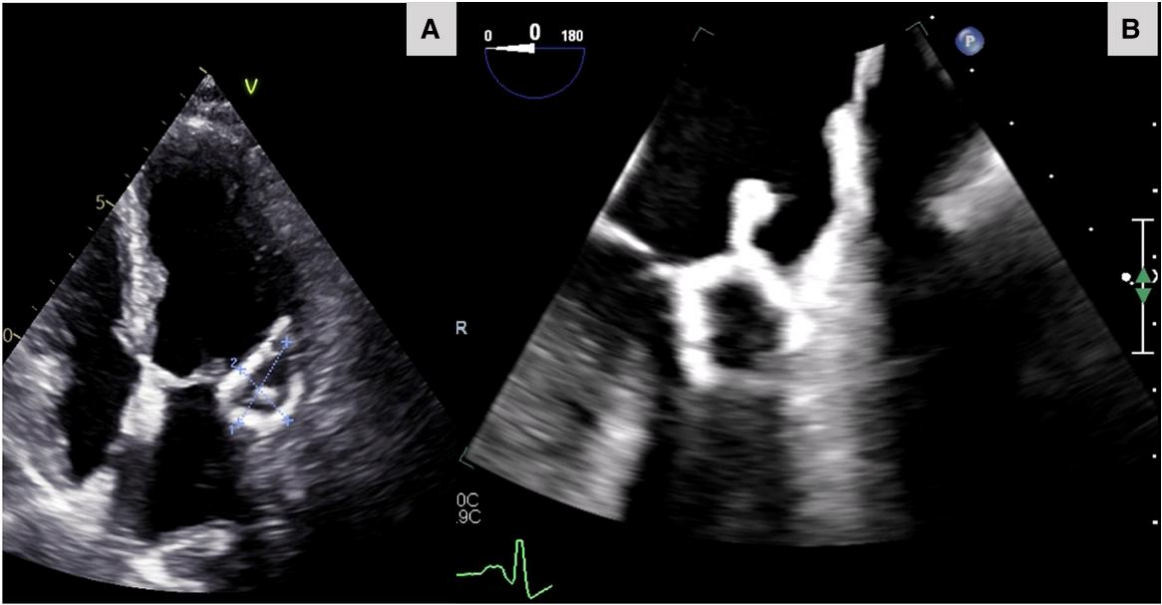
(*A*) Transthoracic echocardiography (apical four-chamber view) demonstrating the posterior mitral annular mass. (*B*) Transoesophageal echocardiography confirming the lesion and revealing a highly mobile atrial-side component.

**Figure 2:**
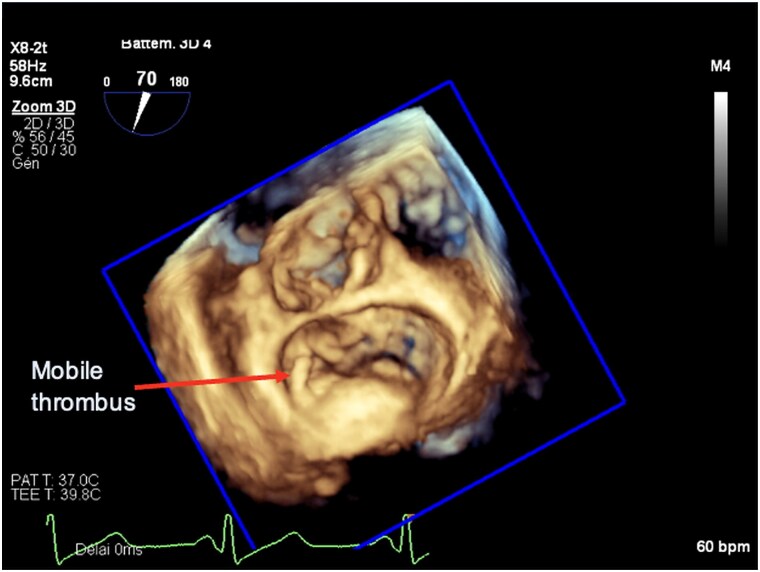
Transoesophageal three-dimensional echocardiography (3D surgical view) showing the mass arising from the posterior mitral annulus with mobile thrombus attached.

**Figure 3:**
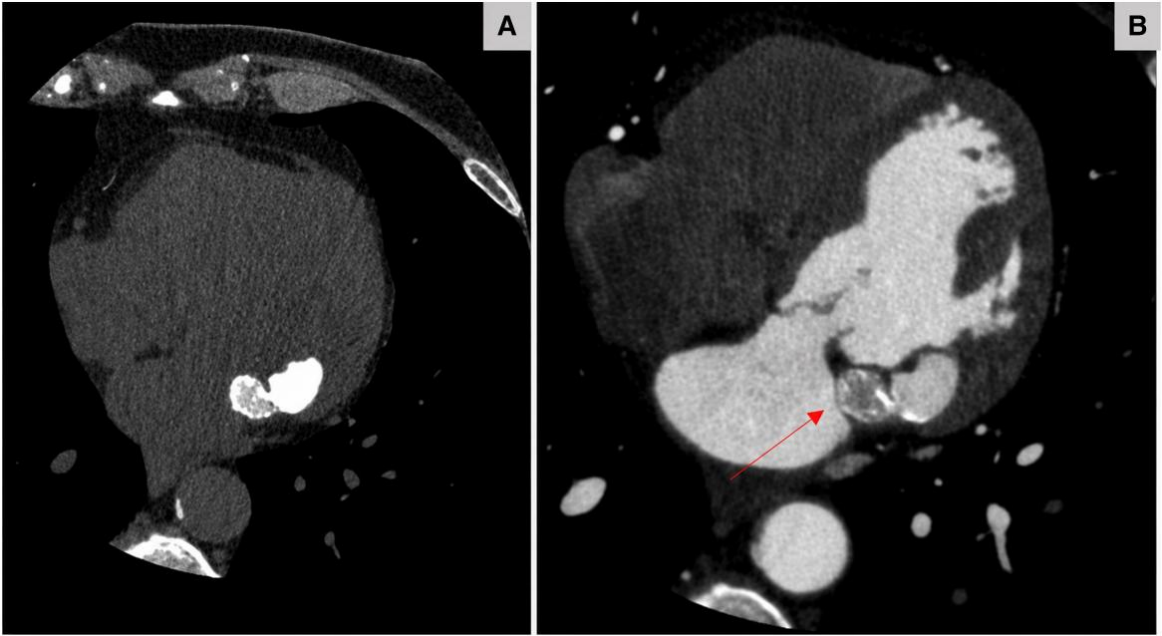
(*A*) Non-contrast CT image showing the marked calcific nature of the posterior mitral annular mass. (*B*) Contrast-enhanced CT showing a well-defined lesion with asymmetric margins and heterogeneous attenuation, including a thin line of calcification (red arrow).

**Figure 4:**
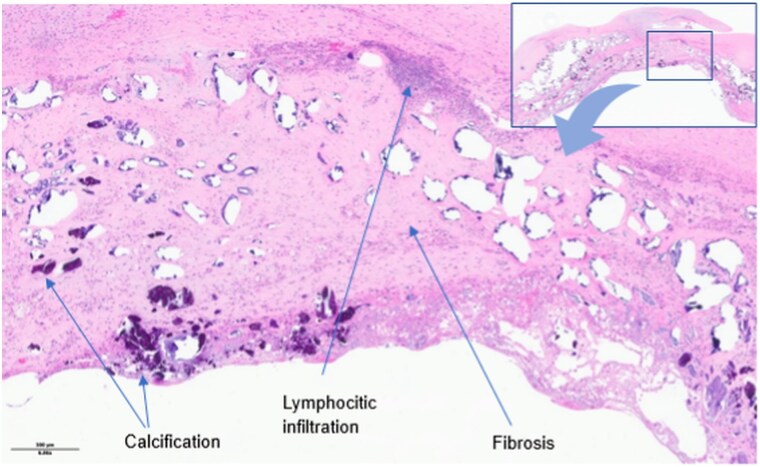
Histopathological analysis of the posterior mitral annular mass. Microscopy demonstrates dense fibrous tissue with widespread calcific deposits and focal ossification. No acute neutrophilic infiltrate suggestive of active infection or features of malignancy are observed.

Serial follow-up echocardiograms (2022–2024) confirmed stable CMAC with persistent moderate mitral regurgitation and no recurrent thrombus. Because of unstable INR values during follow-up, anticoagulation was switched from acenocoumarol to apixaban 5 mg twice daily in April 2022. In 2025, new MRI lesions affecting supra- and infratentorial areas indicated recurrent embolic strokes. TTE and TEE revealed progression of the mass (28 mm × 22 mm × 25 mm) with a mobile atrial component (12 mm), suggesting a high embolic risk (*Figures 1,2*). Repeat aetiological work-up did not identify an alternative embolic source. Prolonged rhythm monitoring did not reveal atrial fibrillation, and carotid as well as intracranial vascular imaging showed no haemodynamically significant stenosis. Blood cultures and inflammatory markers were negative. Cardiac CT excluded obstructive coronary artery disease and confirmed imaging findings consistent with CMAC within extensive posterior mitral annular calcification (*Figure 3*). Following multidisciplinary Heart Team discussion, surgery was recommended because of recurrent embolic strokes despite therapeutic anticoagulation and the development of a highly mobile atrial component attached to the CMAC lesion. Mitral valve replacement was preferred over repair or isolated debridement because of extensive posterior annular involvement, associated posterior leaflet sclerosis and restriction, and persistent moderate mitral regurgitation. The patient underwent mitral valve replacement with a bioprosthesis and left atrial appendage exclusion. The calcified mass was entirely resected. Histopathology showed fibro-inflammatory remodelling with nodular calcification and ossification, consistent with advanced CMAC (*Figure 4*). No definite thrombus was identified on the surgical specimen. Therefore, the mobile echocardiographic component may have represented either superimposed thrombotic material or friable fibro-calcific/caseous material related to the lesion itself. Recovery was uneventful; neurological function improved except for mild cognitive and visual sequelae. Following complete resection of the mitral annular lesion, the embolic substrate was deemed eradicated. In line with standard practice after mitral bioprosthesis implantation, anticoagulation with a vitamin K antagonist was prescribed for three months, followed by transition to a direct oral anticoagulant, with regular echocardiographic monitoring. The patient made a full neurological recovery and remained in excellent general condition at six-month follow-up.

## Discussion

CMAC represents a rare and advanced form of MAC, resulting from dystrophic liquefaction of the calcified annular tissue.^1^ Histologically, it consists of an acellular necrotic core containing lipid, cholesterol deposits, cellular debris, and degradation products surrounded by a calcified fibrous capsule.^4–6^ The exact pathophysiological mechanism remains incompletely understood but is thought to involve chronic degeneration, local inflammation, impaired vascularization, and disturbances of calcium–phosphate metabolism.^5,6^

CMAC predominantly affects elderly women with cardiovascular risk factors and is most often detected incidentally. Although usually benign, it may cause embolic events, valvular dysfunction, or conduction abnormalities.^1,6^ CMAC has also been described in drug-induced valvular heart disease, particularly related to benfluorex, which is no longer in use.^7^

From a diagnostic perspective, CMAC represents a well-recognized pitfall, as it may mimic infective endocarditis, intracardiac tumours, or sterile abscesses.^4,6^ Transthoracic and transoesophageal echocardiography remain first-line modalities and often offer very characteristic features that are diagnostic. In case of diagnostic uncertainty, cardiac CT can identify the characteristic calcified hyperdense shell on non-contrast CT images and a low-density central necrotic core (<50 HU) on post-contrast CT images.^1^ Non-contrast cardiac CT provides superior assessment of CMAC by clearly delineating the hyperdense calcified shell, which may be less conspicuous on contrast-enhanced studies, thereby reducing the risk of misinterpretation as an intracardiac mass. Cardiac MRI can serve as a problem-solving tool, showing a hypointense lesion on T1- and T2-weighted sequences with peripheral late gadolinium enhancement, allowing exclusion of tumoral or infectious masses.^4,8^

Although often considered benign, CMAC may demonstrate dynamic morphological changes over time that can modify embolic risk and clinical presentation. Conservative management is generally appropriate in asymptomatic patients; however, surgical intervention should be considered in the presence of recurrent embolic events, significant valvular dysfunction, or diagnostic uncertainty.^3,9,10^

This approach is consistent with the 2021 ESC/EACTS Guidelines for the management of valvular heart disease and previous reports, which support multidisciplinary Heart Team evaluation and intervention in selected high-risk valvular conditions associated with embolic complications or significant structural abnormalities.^11^ In this patient, the development of a mobile mitral annular component with recurrent embolic strokes despite anticoagulation supported surgical management.

This case illustrates the importance of long-term surveillance of CMAC and highlights the role of multimodality imaging and individualized decision-making in managing rare but potentially serious complications of mitral annular calcification.

## Lead author biography


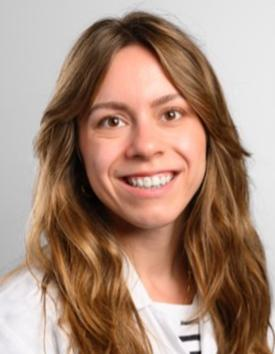


Elsa Hoti is a physician based in Switzerland with clinical experience in cardiac surgery at Lausanne University Hospital (CHUV). She is currently pursuing an MD focused on the impact of mitral valve surgery on ventricular arrhythmias in patients with malignant mitral valve prolapse. Her academic interests lie in valvular heart disease and arrhythmias, with a particular focus on the interface between structural heart disease and electrophysiology. She is aiming to pursue specialist training in cardiology.

## Author contributions

Elsa Hoti (Data curation, Investigation, Writing—original draft [lead]), Jorge Sierra (Validation, Writing—review & editing [equal]), Georgios Tzimas (Resources [supporting], Writing—review & editing [equal]), Francesc Carbo-Descamps (Resources, Writing—review & editing [equal]), and Sarah Hugelshofer (Data curation, Investigation [supporting], Supervision, Writing—review & editing [lead])

## Supplementary Material

ytag471_Supplementary_Data

## Data Availability

The data underlying this article are available in the article and in its online supplementary material.
